# A Rare Case of a Pure Testicular Seminoma Presenting 7 Years after a Pineal Germinoma

**DOI:** 10.1155/2013/490638

**Published:** 2013-10-10

**Authors:** Usman Najeeb Bhatty, Mohammed Hayat Ashrafi, Caroline Mary Nicholson, Ahsanul Haq

**Affiliations:** ^1^Urology Department, Lancashire Teaching Hospitals NHS Trust, Sharoe Green Lane, Fulwood, Preston PR2 9HT, UK; ^2^Histopathology Department, Lancashire Teaching Hospitals NHS Trust, Sharoe Green Lane, Fulwood, Preston PR2 9HT, UK

## Abstract

Pure testicular seminomas occurring in patients with previous intracranial germ cell tumours are extremely rare. We present such a case. A 37-year-old gentleman presented to urology after previously being treated for a pineal germinoma with steroids and radiotherapy. On routine followup, he described symptoms of a testicular seminoma. This was managed surgically with radical orchidectomy. We discuss the possible causes of such an association with a review of the literature.

## 1. Introduction

Germ cell malignancies are common and increasing in males. However, a handful of cases have reported the rare occurrence of testicular germ cell tumours some years after initial extragonadal presentation [1–4]. It is generally thought that the germ cell tumour represents a second primary tumour, as opposed to metastasis. Previous reports have suggested that genetic mutations contribute to their development [5–7]. We present a case of a pure testicular seminoma developing in a patient 7 years after successful treatment of a pineal germinoma.

## 2. Case Report

A thirty-year-old man presented to the outpatient clinic with a two-month history of headaches, nausea, and vomiting. He was previously fit with no relevant family history but was a smoker. Examination revealed bilateral papilloedema, and baseline blood tests showed raised serum prolactin levels and decreased serum cortisol, FSH, and LH levels but normal serum bHCG and AFP levels. 

**Figure 1:**
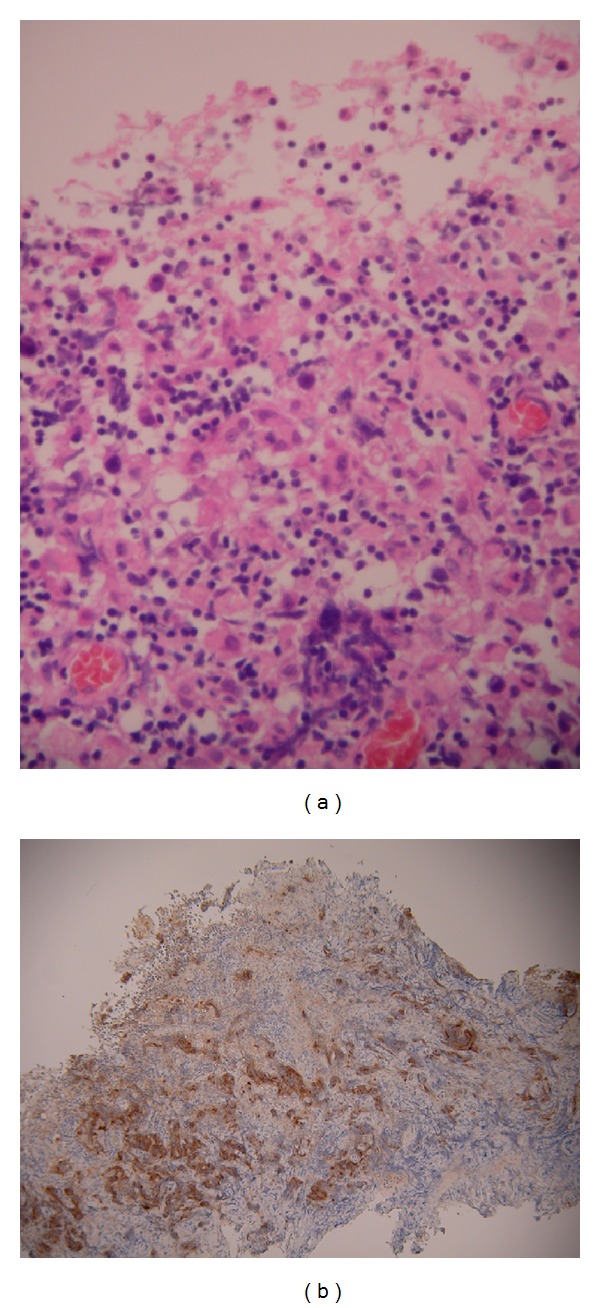
(a) Rather crushed biopsy of the pineal lesion showing lymphocytes surrounding occasional large epithelioid cells. (b) Placental alkaline phosphatase (PLAP) immunohistochemical staining of the pineal lesion showing strong positivity within the crushed epithelioid cells in keeping with a germinoma.

An MRI scan of the head and spine revealed an intracranial mass measuring 3 cm × 3 cm × 3 cm. This mass compressed the cerebral aqueduct resulting in ventriculomegaly. The lesion was a large pineal germinoma confirmed histopathologically containing a small nonenhancing necrotic area (Figures 1(a) and 1(b)). There was no radiological evidence of spinal involvement.

An endoscopic third ventriculostomy was performed to reduce intracranial pressure. A ventriculoperitoneal shunt was avoided because of the risk of intra-abdominal seeding as has been described in the literature [3]. A biopsy of the mass confirmed features in keeping with a germinoma. He was commenced on dexamethasone and underwent 16 fractions of radiotherapy. The patient remained symptom-free and annual surveillance using MRI and repeat serum blood tests showed no evidence of recurrence. 

**Figure 2:**
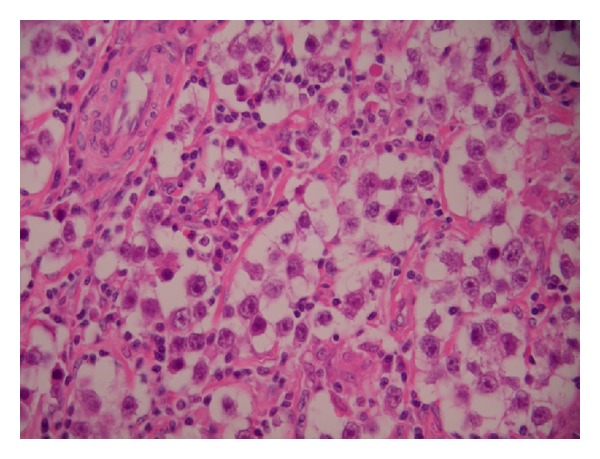
Nests of undifferentiated cells with pale cytoplasm, with a lymphocytic infiltrate typical of classical seminoma.

Seven years later at routine followup, the patient complained of an enlargement of his left testicle. Examination confirmed a hard testicular mass. An ultrasound scan showed a heterogeneous lesion measuring approximately 40 mm × 25 mm × 31 mm, with appearances suggestive of malignancy. An urgent urological opinion was requested, and he subsequently underwent a left inguinal radical orchidectomy. Histology confirmed a classical seminoma (pT1) with no rete invasion (Figure 2). The right testis was normal. A staging CT scan ruled out nodal and distal metastatic disease. Adjuvant chemotherapy was commenced.

Posttreatment, routine PSA level was 4.6 micrograms/litre, but TRUS and prostate biopsies were negative for malignant disease. At 6-month follow up, the patient had fully recovered with no signs of recurrence.

## 3. Discussion

Germ cell tumours are the commonest malignancy in young men, and their prevalence is increasing [6]. Germinomas represent about 60% of all CNS tumours [8]; the pineal gland is a common site of those that are intracranial [1]. To the best of our knowledge, there are only four other reports of a pure testicular seminoma developing some years after a previous pineal germinoma [1–4]. 

All five patients with this combination of disease were initially detected after suffering symptoms of raised intracranial pressure. It may be that some patients treated for testicular seminoma also have a clinically undetectable pineal germinoma. Therefore, although this pattern of disease is rare in the literature, the actual prevalence may be higher.

It was previously understood that extragonadal germ cell tumours represented metastasis of malignant gonadal cells. On the contrary, we believe the testicular seminoma represents a second primary germ cell tumour, in keeping with recent reports. This is likely due to a mutation in the germ cell line. The extensive time interval between the two tumours presenting suggests this. Chan et al. reported a pineal germinoma occurring simultaneously with a testicular seminoma [9]. However, a widespread metastasis from a single primary tumour cannot be ruled out because of the concurrent presentation of both tumours. Alternative theories such as oncogenic factors influencing both gonadal and cranial cells have been suggested [5].

Recently, a genetic basis has been proposed for these tumours. Mutations in the *KIT* gene at codon 816 are associated with gonadal and extragonadal germ cell tumours [6]. Furthermore, it is thought such mutations occur very early during embryogenesis, prior to the migration of germ cells to gonadal and extragonadal locations. Coffey et al. found a *KIT* mutation in a patient who suffered from a pineal germinoma and a testicular seminoma [6]. Other studies have suggested a gene locus on chromosome Xq27 as a possible site for susceptibility to germ cell tumours [7]. One report describes a testicular mixed germ cell tumour arising some years after a pineal germinoma [5]. The authors state the *CCND2* gene as the cause. These reports demonstrate a genetic origin for extragonadal and gonadal germ cell tumours. In order to confirm a genetic aetiology, a family study or genetic analysis would be needed. Despite this, there is increasing evidence to favour a genetic pathophysiology.

There are now a growing number of reports describing an association between intracranial and testicular germ cell tumours, with increasing evidence to confirm a genetic link. We would encourage patients who have suffered a pineal germinoma to perform regular testicular self-examinations [4]. The prognosis following pineal germinoma is very good with a reported 5-year survival rate above 90% [10]. Therefore, there should be a greater awareness amongst urologists of a possible long-term association with testicular seminomas. Genetic studies into concurrent pineal germinomas and testicular seminomas are still needed.
